# Establishment of a Sandwich ELISA for Detection of Pan-Merbecoviruses

**DOI:** 10.3390/pathogens14060605

**Published:** 2025-06-19

**Authors:** Kaixin Li, Misa Katayama, Ayano Ichikawa, Hiromichi Matsugo, Yuta Wakabayashi, Akiko Takenaka-Uema, Wataru Sekine, Taisuke Horimoto, Shin Murakami

**Affiliations:** 1 Laboratory of Veterinary Microbiology, Graduate School of Agricultural and Life Sciences, University of Tokyo, Tokyo 113-8657, Japan; haydenlai0718@g.ecc.u-tokyo.ac.jp (K.L.); mfj-ktym@g.ecc.u-tokyo.ac.jp (M.K.); ichi11-11-1@g.ecc.u-tokyo.ac.jp (A.I.); matsugo-hiromichi@g.ecc.u-tokyo.ac.jp (H.M.); wakabayashi-yuta634@g.ecc.u-tokyo.ac.jp (Y.W.); atakiko@g.ecc.u-tokyo.ac.jp (A.T.-U.); w-sekine@g.ecc.u-tokyo.ac.jp (W.S.); 2 Laboratory of Veterinary Public Health, Graduate School of Agricultural and Life Sciences, University of Tokyo, Tokyo 113-8657, Japan

**Keywords:** bat merbecoviruses, sandwich ELISA, antigen detection, monoclonal antibody

## Abstract

*Merbecovirus*, a subgenus of *Betacoronavirus*, includes MERS-CoV and multiple bat-derived viruses with zoonotic potential. Given the unpredictable emergence of these viruses and their genetic diversity, development of broad-spectrum diagnostic tools is expected. In this study, we established a sandwich ELISA targeting the nucleocapsid (N) protein of merbecoviruses. We generated monoclonal antibodies (mAbs) using recombinant N protein of a bat merbecovirus, VsCoV-1, and selected cross-reactive clones for other merbecoviruses. Three mAbs showed strong reactivities with multiple merbecoviruses but not with SARS-CoV-2 or endemic human coronaviruses. Pairwise ELISA screening identified 1A8/10H6 mAbs as the optimal combination for detection of N protein from six merbecoviruses—VsCoV-1, EjCoV-3, MERS-CoV, NeoCoV, HKU4, and HKU5—with limits of detection (LODs) below 7.81 ng/mL, including 1.25 ng/mL for VsCoV-1. Infectious bat merbecovirus EjCoV-3 was detected at 1.3 × 10^3^ PFU/mL. No cross-reactivity was observed with non-merbecoviruses, indicating its high specificity. This sandwich ELISA offers a rapid, reproducible, and cost-effective diagnostic platform with potential for high-throughput screening and automation. Moreover, its design is amenable to adaptation into point-of-care formats such as lateral flow assays, highlighting its value for field-based surveillance and pandemic preparedness.

## 1. Introduction

Coronaviruses from the *Betacoronavirus* genus of the family *Coronaviridae* have caused three major outbreaks over the past two decades: severe acute respiratory syndrome (SARS), Middle East respiratory syndrome (MERS) [1], and COVID-19 [2], all of which have posed significant public health challenges [3,4]. It is believed that these pathogenic viruses originate from bats [5]. Bats harbor a wide variety of coronaviruses, including betacoronaviruses, and the emergence of novel coronavirus infections from bat-derived betacoronaviruses poses a concern [6,7]. Therefore, in preparation for potential outbreaks of such infections, the development of preventive measures, treatments, and diagnostic methods is critical.

**Figure 1:**
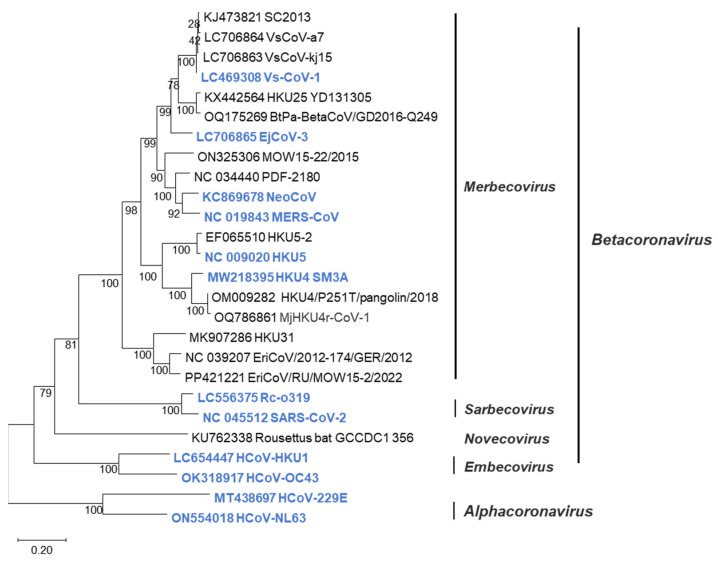
Phylogenetic tree of merbecoviruses. Phylogenetic trees were generated using the N gene sequences of coronaviruses via maximum-likelihood analysis combined with 1000 bootstrap replicates. Bootstrap values are indicated in the major nodes. Scale bars indicate nucleotide substitutions per site. Viruses colored blue were tested in sandwich ELISA.

Betacoronavirus is classified into four subgenera: *Merbecovirus*, *Sarbecovirus*, *Embecovirus*, and *Nobecovirus* [8]. The merbecoviruses are further classified into MERS coronavirus (MERS-CoV) clade; bat-associated clades such as HKU4 [9], HKU5 [10], HKU25 [11], and MOW [12]; and hedgehog-associated EriCoV clade [13] (Figure 1). These merbecoviruses are genetically diverse, and many of them possess the ability to infect human cells [14,15], suggesting their potential to cause novel respiratory infections in humans. However, as it is currently unpredictable which specific merbecovirus might trigger an outbreak in humans, there is an urgent need to develop diagnostic methods capable of detecting various merbecoviruses.

The sandwich ELISA method is a highly sensitive and specific assay for detecting viral antigens. It utilizes a dual-antibody system that minimizes nonspecific binding and enables quantitative analysis through colorimetric measurement. Compared to RT-PCR, which requires specialized equipment and technical expertise, or virus isolation, which can be time-consuming and labor-intensive, sandwich ELISA offers a faster, simpler, and more cost-effective alternative [16]. To further illustrate these advantages, we have included a Supplementary Table (Table S1) comparing key practical aspects—such as cost per test, assay time, and technical requirements—between our sandwich ELISA and standard RT-PCR–based methods. Sandwich ELISA supports high-throughput screening, provides strong reproducibility, and is easily automated, making it ideal for standardized diagnostics. Furthermore, the antibody pair of sandwich ELISA can be applied to immunochromatographic assays, enabling the development of rapid, user-friendly point-of-care tests without sacrificing accuracy. Thus, sandwich ELISA serves as both a reliable laboratory diagnostic tool and a foundation for advancing practical, field-ready diagnostic technologies.

The N protein of coronaviruses is the most abundant protein in the viral particle. Among the merbecoviruses, it exhibits a relatively high homology [17]. Therefore, it is an ideal target for antigen-detection ELISA. In this study, we generated a pair of mouse monoclonal antibodies capable of detecting the N protein in a wide range of merbecoviruses and established a sandwich ELISA system for N protein detection.

## 2. Materials and Methods

### 2.1. Cells

Human embryonic kidney 293T cells (Lenti-X; Takara Bio, Shiga, Japan) and Vero cells stably expressing transmembrane serine protease 2 (Vero/TMPRSS2), which were kindly provided by Dr. Makoto Takeda (National Institute of Infectious Diseases), were maintained in Dulbecco’s modified Eagle’s medium (DMEM; Fujifilm Wako Pure Chemical, Osaka, Japan) supplemented with 10% fetal bovine serum (FBS; Nichirei, Tokyo, Japan). Madin–Darby canine kidney (MDCK) cells (ATCC CCL-34) were maintained in Eagle’s minimum essential medium (MEM; Life Technologies, Carlsbad, CA, USA) supplemented with 5% newborn bovine serum (NBS). Mouse myeloma P3X63Ag8U.1 (P3U1) cells (JCRB0708; NIBIOHN, Osaka, Japan) were cultured in RPMI-1640 medium (Fujifilm Wako) supplemented with 10% FBS (Sigma, Burlington, MA, USA). All cell lines were incubated at 37 °C in a 5% CO_2_ atmosphere.

### 2.2. Viruses

The bat merbecovirus EjCoV-3 (GenBank accession no. LC706865) [18], identified in *Eptesicus japonensis*, was used as the virus sample. Infectious EjCoV-3 was generated using a reverse genetics system in which the full genome was cloned into a bacterial artificial chromosome (BAC) to create the EjCoV-3 BAC clone [19]. The rescued virus was propagated in Vero/TMPRSS2 cells in DMEM containing 1% FBS and 5 µg/mL thermolysin (Nacalai Tesque, Kyoto, Japan), aliquoted, and stored at −80 °C. Virus titers were determined by plaque assay. Confluent Vero/TMPRSS2 cells in 12-well plates were infected with 10-fold serial dilutions of EjCoV-3 in DMEM with 1% FBS and 5 µg/mL thermolysin. After 1 h at 37 °C, cells were washed and overlaid with MEM containing 1% FBS, 0.8% Seakem GTG agarose (Lonza Japan, Chiba, Japan), and 5 µg/mL thermolysin. Plates were incubated for 2 days at 37 °C, 5% CO_2_. Plaques were visualized using 0.1% crystal violet in methanol after removal of the agarose overlay. Titers were expressed as plaque-forming units (PFUs). Human coronaviruses NL63, HKU1, OC43, and 229E [20] were kindly provided by Dr. Yohei Matoba (Yamagata Prefectural Institute of Public Health).

### 2.3. Plasmids

N gene sequences of VsCoV-1 (LC469308.1) [18], MERS-CoV (NC_019843.3), EjCoV-3, HKU5 (NC_009020.1), HKU4 (MW218395.1), NeoCoV (KC869678.4) [20], SARS-CoV-2 (NC_045512.2), Rc-o319 (LC556375.1) [21], and human coronaviruses NL63, HKU1, OC43, and 229E [20] were obtained from various sources. RNA was extracted from bat feces (VsCoV-1) or virus-containing culture supernatants (EjCoV-3, SARS-CoV-2, Rc-o319, NL63, HKU1, OC43, and 229E) using the NucleoSpin^®^ VET kit (Macherey-Nagel, NRW, Düren, Germany). For RNA-derived templates, cDNA was synthesized using oligo(dT) primers and PrimeScript™ II Reverse Transcriptase (Takara Bio), followed by PCR with KOD FX Neo (Toyobo, Osaka, Japan). N gene sequences of MERS-CoV, HKU5, HKU4, and NeoCoV were synthesized by Eurofins Genomics (Tokyo, Japan).

All N genes were cloned into pCAGGS-based mammalian expression vectors via EcoRI and XhoI sites using the NEBuilder HiFi DNA assembly kit (New England Biolabs, Ipswich, MA, USA). To express GST-tagged N proteins, the same sequences were cloned into pGEX-6P-1 vectors digested with BamHI and EcoRI, which contain a PreScission protease cleavage site. The resulting constructs included pGEX-VsCoV-1-N, pGEX-MERS-N, pGEX-EjCoV-3-N, pGEX-HKU5-N, pGEX-HKU4-N, pGEX-NeoCoV-N, and pGEX-SARS-CoV-2-N.

### 2.4. Preparation of Recombinant N Proteins

**Figure 2:**
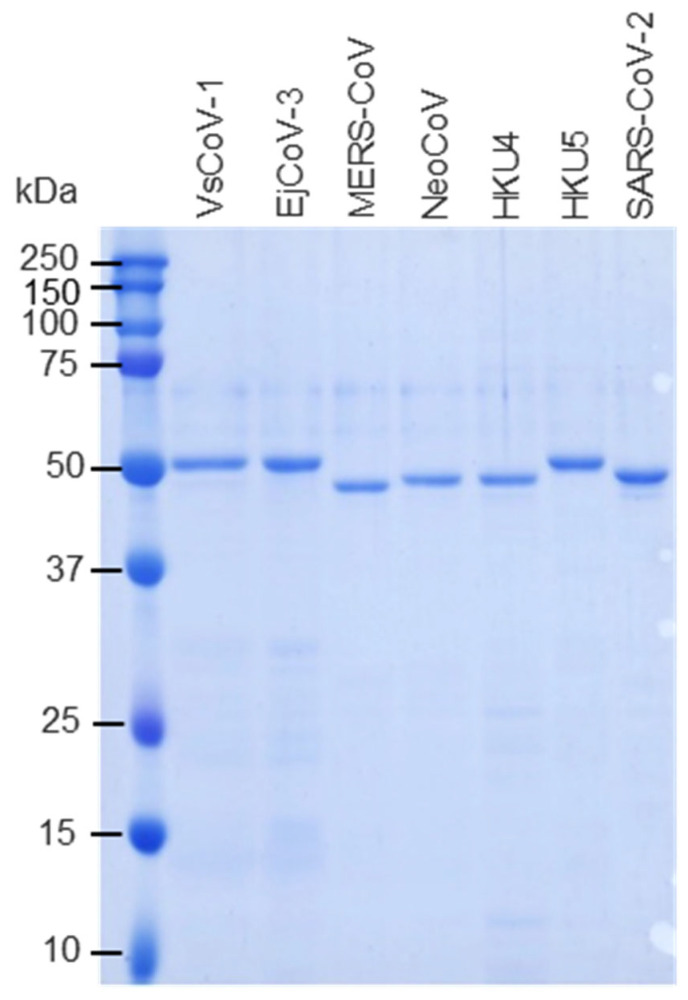
Expression and purification of recombinant merbecovirus proteins. GST-tagged merbecovirus N proteins were expressed in *E. coli*. The proteins were purified using glutathione affinity column and the GST-tags were removed by digestion of PreScission proteases. The purities of recombinant proteins were subjected to SDS-PAGE.

The above pGEX constructs were individually transformed into *Escherichia coli* BL21 cells. Ampicillin-resistant clones were cultured in LB medium, and protein expression was induced with 0.1 mM IPTG at 25 °C for 12 h. Cells were lysed in PBS containing 1% Triton X-100 (Polysciences, Warrington, PA, USA), ultra-sonicated, and centrifuged at 10,000× *g*. Soluble fractions were purified using Glutathione Sepharose™ 4B resin (Cytiva, Marlborough, MA, USA). GST tags were removed by PreScission protease (Cytiva), and protein concentrations were measured using a BCA protein assay kit (Takara Bio). Protein purities were assessed by SDS-PAGE (Figure 2).

### 2.5. Generation of Monoclonal Antibodies Against VsCoV-1 N Protein

**Figure 3:**
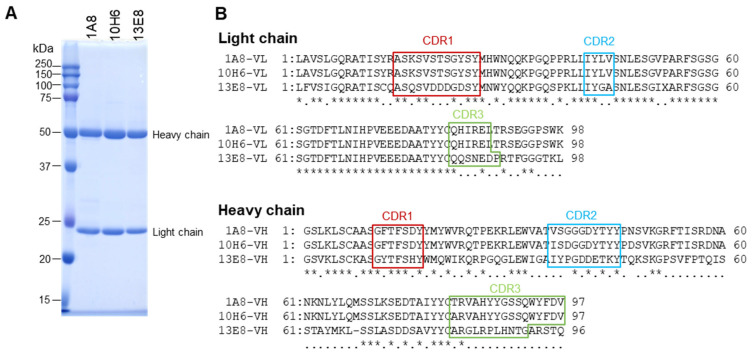
Characterization of monoclonal antibodies (mAbs). (**A**) Purified mAbs obtained using Protein L affinity chromatography were analyzed by SDS-PAGE. (**B**) Amino acid sequences of the variable regions of the mAbs were determined by Sanger sequencing. Asterisks (*) indicate positions with identity among all three aligned sequences, while dots (·) indicate identity between two of the three.

BALB/c mice (4-week-old females, Japan SLC, Shizuoka, Japan) were immunized subcutaneously with 50 µg of purified rVsCoV-1 N protein in TiterMax Gold adjuvant (Titer Max, Norcross, GA, USA) three times at two-week intervals. Mice with high ELISA titers received a final intravenous booster and were euthanized three days later. Splenocytes were fused with P3U1 cells using polyethylene glycol, and hybridomas were screened by ELISA using rVsCoV-1 N as an antigen. Positive clones were subcloned and injected into pristane-primed mice for ascites production. Monoclonal antibodies (mAbs) were purified by ammonium sulfate precipitation, dialysis, and affinity chromatography with Protein A or L (GL Sciences, Tokyo, Japan), depending on isotype (determined by IsoStrip kit, Roche Diagnostics, Indianapolis, IN, USA). The mAb purities were assessed by SDS-PAGE (Figure 3A).

Cross-reactivity was assessed by indirect immunofluorescence assay (IFA) [22]. MDCK cells were transfected with pCAGGS-based N plasmids for 12 coronaviruses using PEI (Polysciences, Warrington, PA, USA). After 24 h, cells were fixed with 4% paraformaldehyde, permeabilized, and incubated with each mAb. Alexa Fluor 488-conjugated goat anti-mouse IgG was used for detection, and fluorescence was observed with an Axio Vert.A1 microscope (ZEISS, Oberkochen, Germany).

For sequence analysis, total RNA was extracted from hybridomas using Isogen reagent (Nippon Gene, Tokyo, Japan). cDNA was synthesized using chain-specific primers, and variable regions of heavy and light chains were amplified by PCR and Sanger sequenced.

### 2.6. Development and Optimization of a Sandwich ELISA for Detection of Merbecoviruses

Monoclonal antibodies were labeled with horseradish peroxidase (HRP) using HRP conjugating kit (Dojindo, Kumamoto, Japan). A sandwich ELISA was developed using recombinant VsCoV-1 N and EjCoV-3 virus lysate as antigens. EjCoV-3 was inactivated and lysed in RIPA buffer (150 mM NaCl, 50 mM Tris-HCl pH 8.0, 2 mM EDTA, 1% Triton X-100, 0.5% SDC, 0.1% SDS) at room temperature for 30 min. Samples were diluted in PBS-T as needed.

Microplates were coated with cross-reactive mAbs (0.5 µg/mL) in bicarbonate buffer (pH 9.6) overnight at 4 °C. After blocking with 7.5% BSA, rVsCoV-1 N or EjCoV-3 lysate were applied as test antigens. PBS-T and mock-infected lysate served as negative controls for rVsCoV-1 N or EjCoV-3 lysate, respectively. HRP-conjugated mAbs were used for detection. Color was developed with TMB substrate, stopped with 1 N H_2_SO_4_, and absorbance was measured at 450 nm. Optimal antibody pairs were selected based on signal-to-background (S/B) ratios262728. Antibody concentrations were optimized by titration (1–8 µg/mL for capture, 0.5–4 µg/mL for detection).

### 2.7. Evaluation of Sandwich ELISA Performance

The cut-off value was defined as the mean OD at 450 nm of the negative control plus three standard deviations. The specificity was assessed using lysates from 293T cells transfected with plasmids encoding the N genes of 12 different coronaviruses: VsCoV-1, EjCoV-3, MERS-CoV, NeoCoV, HKU4, HKU5, SARS-CoV-2, Rc-o319, HCoV-OC43, HCoV-HKU1, HCoV-229E, and HCoV-NL63, as well as a control pcDNA-Venus vector.

The limit of detection (LOD) for each virus was estimated using a linear approximation method. Two-fold serial dilutions of recombinant N proteins (500–7.8125 ng/mL) described above were analyzed by sandwich ELISA, and the OD was plotted against the logarithm of antigen concentrations.

### 2.8. Detection Limit of Sandwich ELISA

Recombinant VsCoV-1 N protein (rVsCoV-1-N) was two-fold serially diluted from 80 ng/mL to 0.675 ng/mL, and infectious EjCoV-3 was five-fold serially diluted from 2.0 × 10^6^ to 2.6 × 10^2^ PFU/mL (n = 3). Each dilution was used as an antigen in the sandwich ELISA. For rVsCoV-1-N, PBS-T (100 µL/well) served as the negative control. For EjCoV-3, DMEM containing 1% fetal bovine serum (FBS) (100 µL/well) was used as the negative control. The detection limit (LOD) was determined using a cut-off value defined as the mean of the negative control signals plus three standard deviations (X + 3SD).

### 2.9. Ethics Statement

All animal experiments were conducted in accordance with the Regulations for Animal Care at the University of Tokyo and were approved by the Animal Experiment Committee of the Graduate School of Agricultural and Life Sciences, University of Tokyo. No in vivo infection experiments or survival analyses were performed in this study.

## 3. Results

### 3.1. Generation and Characterization of Monoclonal Antibodies

The purified rVsCoV-1-N protein was used as an antigen for monoclonal antibody generation in mouse and for subsequent ELISA development. Ten hybridoma cell lines producing anti-rVsCoV-1-N monoclonal antibodies (mAbs) were established. Isotypes were determined using an isotyping kit (Table 1). To assess cross-reactivity, indirect immunofluorescence assays (IFAs) were performed using MDCK cells transfected with pCAGGS-based plasmids expressing N proteins of various merbecoviruses and other coronaviruses. Three mAbs (1A8, 10H6, and 13E8) exhibited strong reactivity with merbecoviruses (VsCoV-1, EjCoV-3, MERS-CoV, NeoCoV, HKU4, HKU5) but not with SARS-CoV-2, Rc-o319, HKU1, OC43, 229E, or NL63 (Table 2). These three mAbs were selected for further ELISA development, propagated in mouse ascites, and purified (Figure 3A).

Sanger sequencing of the variable regions revealed that 1A8 and 10H6 shared identical light chains and highly similar heavy chains (four amino acid differences), whereas 13E8 displayed distinct sequences in both chains (Figure 3B). These data suggest that 1A8 and 10H6 recognize an identical or very closely related epitope.

### 3.2. Selection of Antibody Pairs for Sandwich ELISA

**Figure 4:**
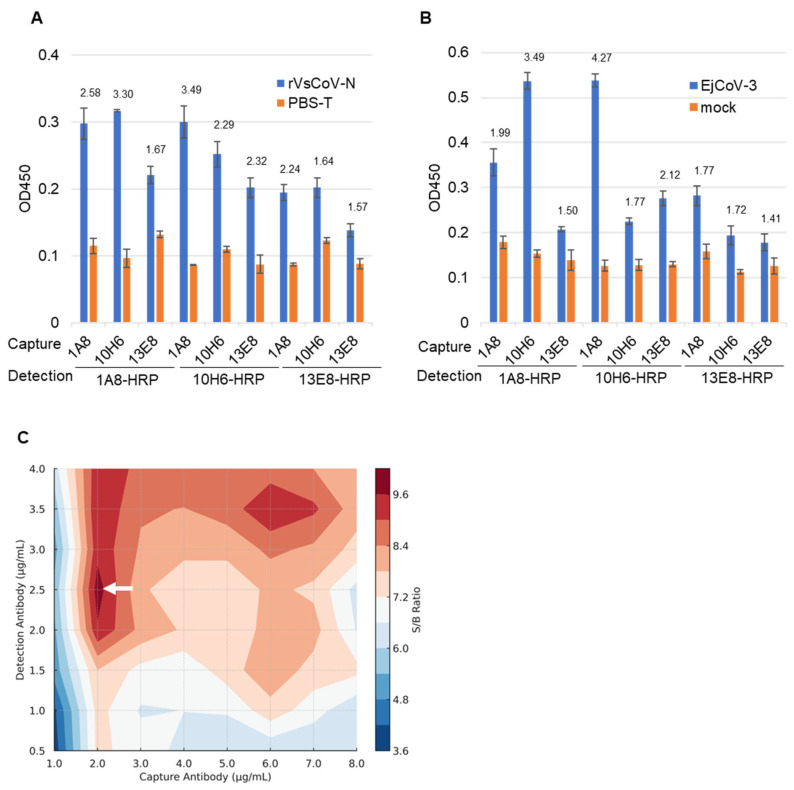
Optimization of the sandwich ELISA antibody pair combinations. The bar graph represents the sandwich ELISA absorbance values (OD 450) obtained from different mAb pair combinations. The capture mAbs (13E8, 1A8, 10H6) and HRP-conjugated detection mAbs (13E8-HRP, 1A8-HRP, 10H6-HRP) were tested using rVsCoV-1-N (500 ng/mL) (**A**) and EjCoV-3-infected cell lysate (1:100 dilution) (**B**). Bars indicate the mean absorbance values (OD 450) (n = 3). The signal-to-background (S/B) ratio for each mAb pair is annotated above the respective bars. (**C**) A contour plot illustrates the signal-to-background (S/B) ratio across various combinations of 1A8 and 10H6-HRP concentrations. Higher S/B ratios (shown in white arrow) indicate better assay performance.

Purified mAbs (1A8, 10H6, 13E8) and their HRP-conjugated forms (1A8-HRP, 10H6-HRP, 13E8-HRP) were evaluated in pairwise combinations for sandwich ELISA. Using either rVsCoV-1-N (100 ng/mL) (Figure 4A) or EjCoV-3 lysate (1:100 dilution) (Figure 4B) as antigen, the antibody pairs were screened based on signal-to-background (S/B) ratios. For rVsCoV-1-N detection, the pair of 1A8 (capture) and 10H6-HRP (detection) yielded the highest S/B ratio of 3.49, followed by the reciprocal pair (10H6/1A8-HRP) with a ratio of 3.30. The homologous pairs of 1A8/1A8-HRP and 10H6/10H6-HRP also showed relatively high S/B values (2.59 and 2.28, respectively), whereas combinations involving 13E8 performed poorly. EjCoV-3 lysate showed the same trend as rVsCoV-1-N, with the 1A8/10H6-HRP pair exhibiting the best performance (S/B = 4.27) and the reciprocal pair 10H6/1A8-HRP also yielding a strong signal (S/B = 3.49), further confirming the robustness of this antibody combination across different merbecovirus antigens. The optimal antibody pair—1A8 (capture) and 10H6-HRP (detection)—was selected for the sandwich ELISA. Notably, this antibody pair shares high sequence similarity, which is generally considered suboptimal for sandwich ELISA due to potential epitope overlap. The effectiveness of this combination was therefore unexpected. Antibody concentrations were optimized via titration which ranged from 1 to 8 µg/mL for capture and from 0.5 to 4 µg/mL for detection. A contour plot of S/B ratios indicated that 2 µg/mL of 1A8 and 2.5 µg/mL of 10H6-HRP yielded the best performance (Figure 4C).

### 3.3. Performance Evaluation of the Sandwich ELISA

**Figure 5:**
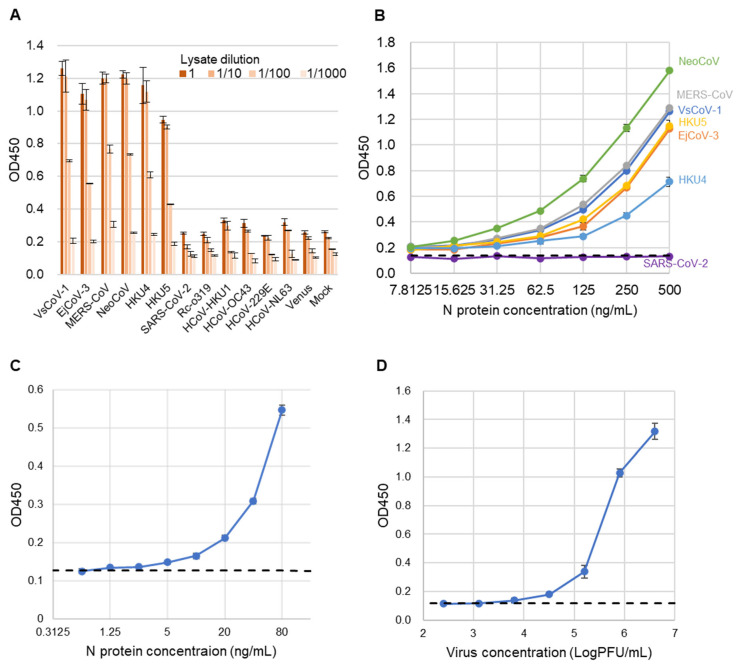
Evaluation of sandwich ELISA for merbecovirus detection. (**A**) ELISA absorbance values (OD 450) for lysates from various coronavirus-expressing cells. Bars indicate mean ± SD (n = 3). Lysates were tested at dilutions of 1, 1/10, 1/100, and 1/1000. (**B**) ELISA absorbance values for purified recombinant N proteins of various coronaviruses diluted from 500 to 7.8125 ng/mL (2-fold). Bars represent mean values (n = 3). PBS-T served as the negative control. The dashed line shows the cut-off value, defined as the mean of PBS-T plus 3 SD. (**C**) Detection limit determination for rVsCoV-1-N. The x-axis shows antigen concentration (ng/mL), and the y-axis indicates absorbance (OD 450). The dashed line represents the cut-off value (PBS-T mean + 3 SD). (**D**) Detection sensitivity for infectious EjCoV-3 virus lysate. The x-axis shows dilution factors, and the y-axis shows absorbance (OD 450). The dashed line indicates the cut-off value (mock-infected control mean + 3 SD).

To assess the specificity of the sandwich ELISA, lysates from 293T cells expressing the N proteins of various coronaviruses were used as antigens (Figure 5A). Positive signals were detected exclusively for viruses belonging to the merbecovirus group. No cross-reactivity was observed with SARS-CoV-2, Rc-o319, HKU1, OC43, NL63, 229E, or the mock control, indicating the high specificity of the assay.

Next, to evaluate the analytical sensitivity of the developed sandwich ELISA, recombinant N proteins from multiple merbecoviruses and SARS-CoV-2 (as a control) were serially diluted and tested (Figure 5B). The assay exhibited comparable analytical sensitivity across all tested merbecoviruses, with the exception of SARS-CoV-2. The estimated limits of detection (LODs) were below 7.815 ng/mL for NeoCoV, MERS-CoV, VsCoV-1, HKU5, HKU4, and EjCoV-3. The exact LOD for rVsCoV-1-N was determined to be 1.25 ng/mL (Figure 5C). Additionally, when using infectious EjCoV-3 virus, the LOD was calculated to be 1.3 × 10^3^ PFU/mL (Figure 5D). These results demonstrate that the assay is broadly applicable for the sensitive detection of a wide range of merbecoviruses.

## 4. Discussion

In this study, we developed a novel sandwich ELISA targeting the N protein of bat-derived merbecoviruses, including those closely related to MERS-CoV. The specificity of the assay was ensured by selecting monoclonal antibodies that react exclusively with merbecoviruses and not with other human coronaviruses. The assay demonstrated high sensitivity and specificity, being capable of detecting recombinant VsCoV-1 N protein at 1.25 ng/mL and EjCoV-3 virus at 1.3 × 10^3^ PFU/mL. These values suggest the potential applicability of this assay in clinical diagnostics and public-health surveillance for emerging merbecoviruses.

Previous studies have reported that viral-antigen sandwich ELISAs exhibit variable detection limits depending on the virus and assay format—for example, 0.04–1 ng/mL for SARS-CoV-2 with sensitivities of approximately 50–70% and specificities approaching 100% [22,23,24,25], and approximately 250 ng/mL for CCHFV [26]. In comparison, our sandwich ELISA demonstrated detection limits below 7.81 ng/mL for recombinant N proteins from various merbecoviruses, including 1.25 ng/mL for rVsCoV-1 and 1.3 × 10^3^ PFU/mL when using infectious virus. These results indicate that the assay is sufficiently sensitive to detect moderate to high viral loads. A limitation of this study is that infectious virus-based sensitivity was evaluated only for EjCoV-3. Future studies including other live merbecoviruses will be necessary to further substantiate the pan-merbecovirus detection capability. This study focused on analytical validation using standardized viral preparations under laboratory conditions. While three independent experiments confirmed the assay’s reproducibility, further studies incorporating a broader range of clinical or biological samples will be necessary to fully evaluate diagnostic applicability in real-world settings. To further evaluate its clinical applicability, validation using clinical specimens is warranted.

In this study, the monoclonal antibodies 1A8 and 10H6 were found to have identical light chain sequences and highly similar heavy chain sequences, the latter differing by only four amino acids, suggesting that they recognize the same or a very closely related epitope. Generally, sandwich ELISA requires antibody pairs that bind to distinct epitopes to allow both capture and detection of the antigen. However, in our study, the 1A8/10H6 pair showed the highest performance despite the epitope similarity. This apparent exception can be explained by the nature of the nucleocapsid (N) protein, which forms ribonucleoprotein complexes with viral genomic RNA or host RNA. In such complexes, multiple N protein molecules are assembled into a higher-order structural unit. Within this multimeric structure, antibodies recognizing similar or overlapping epitopes may simultaneously bind to different N protein molecules within the same complex, thereby enabling effective sandwich detection even in the absence of epitope divergence. This observation challenges the conventional notion that distinct epitopes are strictly required for sandwich ELISA and suggests a novel exception driven by viral protein multimerization. To our knowledge, such a mechanism has not been clearly demonstrated in previous ELISA studies, highlighting the originality of our finding. If confirmed through further structural or epitope-mapping studies, this strategy may also be applicable to other viral antigens with multimeric architecture.

To further enhance its applicability in the field, we propose adapting this sandwich ELISA into an immunochromatographic format. This platform, widely used for the diagnosis of diseases such as COVID-19 [27] and influenza, provides a practical diagnostic solution in resource-limited settings. Transitioning our assay into an immunochromatographic format would allow for portable and large-scale detection of merbecoviruses, making it well-suited for outbreak surveillance and rapid response in high-risk regions.

In conclusion, our sandwich ELISA provides a powerful tool for detecting a wide range of merbecoviruses and holds great promise for clinical application. Recent studies have reported the identification and isolation of several novel merbecoviruses, including those from previously unrecognized hosts such as hedgehogs. While further validation is needed to determine whether our assay is applicable to these newly identified lineages, the broad cross-reactivity and high analytical sensitivity demonstrated in this study suggest that our ELISA system is a highly effective platform for merbecovirus surveillance. In the present study, the N protein of the MERS-CoV EMC strain, a representative clade A virus, was used to evaluate assay performance. Given the high conservation of the nucleocapsid protein among MERS-CoV strains isolated from both dromedary camels and humans, it is likely that the developed ELISA can detect a broad range of MERS-CoV genotypes regardless of host species. However, because minor sequence variations may influence antibody binding, especially among regional or host-specific variants, further validation using clinical isolates from multiple genotypes and vector species is necessary to confirm the assay’s universality. In future studies, we aim to validate this ELISA using clinical specimens from suspected merbecovirus cases in humans. Key steps will include optimizing the assay for respiratory or serum samples, assessing cross-reactivity in clinical matrices, and evaluating diagnostic performance in comparison to gold-standard PCR-based methods. Furthermore, miniaturization of the assay into a point-of-care format will enhance its applicability in field settings. These efforts will be crucial for regulatory approval, clinical deployment, and pandemic preparedness.

## Figures and Tables

**Table 1:** Subtypes of mAbs established in this study.

	mAb									
	1A8	2B4	2H5	3B4	5C11	5F3	7D5	10H6	12F6	13E8
Heavy chain	IgG1	IgG2b	IgG2a	IgG1	IgG2a	IgG2a	IgG1	IgG1	IgG1	IgG1
Light chain	κ	κ	κ	κ	Κ	Λ	κ	Κ	κ	κ

**Table 2:** Cross-reactivity of mAbs with merbecoviruses evaluated by indirect immunofluorescence assay.

	Virus											
mAb	VsCoV	EjCoV	MERS	NeoCoV	HKU4	HKU5	SARS-CoV-2	Rc-o319	HKU1	OC43	229E	NL63
1A8	+	+	+	+	+	+	−	−	−	−	−	−
2B4	+	+	+	−	−	−	−	−	−	−	−	−
2H5	+	−	−	−	−	−	−	−	−	−	−	−
3B4	+	+	+	+	−	−	−	−	−	−	−	−
5C11	+	+	+	−	−	−	−	−	−	−	−	−
5F3	+	+	+	−	−	−	−	−	−	−	−	−
7D5	+	+	−	−	−	−	−	−	−	−	−	−
10H6	+	+	+	+	+	+	−	−	−	−	−	−
12F6	+	+	+	+	+	−	−	−	−	−	−	−
13E8	+	+	+	+	+	+	−	−	−	−	−	−

+ indicates IFA positive; − indicates IFA negative.

## Data Availability

The original contributions presented in this study are included in the article. Further inquiries can be directed to the corresponding author.

## Supplementary Materials

The following supporting information can be downloaded at: https://www.mdpi.com/article/10.3390/pathogens14060605/s1, Table S1: Comparison between sandwich ELISA and RT-PCR for merbecovirus detection.

## Abbreviations

The following abbreviations are used in this manuscript:

ELISA	Enzyme-linked immunosorbent assay
CoV	Coronavirus
mAb	Monoclonal antibody
N	Nucleocapsid
LOD	Limit of detection
SARS	Severe acute respiratory syndrome
MERS	Middle East respiratory syndrome
COVID-19	Coronavirus disease 2019
RT-PCR	Real-time polymerase chain reaction
DMEM	Dulbecco’s modified Eagle’s medium
FBS	Fetal bovine serum
MDCK	Madin–Darby canine kidney cell
MEM	Eagle’s minimum essential medium
NBS	Newborn bovine serum
BAC	Bacterial artificial chromosome
PFU	Plaque-forming unit
HRP	Horseradish peroxidase
PEI	Polyethyleneimine
SD	Standard deviation
IFA	Indirect immunofluorescence assay
S/B	Signal-to-background ratio
CCHFV	Crimean–Congo hemorrhagic fever virus
