# Supplementary material for: Establishment of a Sandwich ELISA for Detection of Pan-Merbecoviruses
Source: Pathogens. 2025 Jun 19;14(6):605. doi: 10.3390/pathogens14060605 (PMC12196402; doi:10.3390/pathogens14060605)
Supplement: Supplementary file 1 [file pathogens-14-00605-s001.zip › pathogens-3677378-supplementary.pdf]

Supplementary Table S1. Comparison between sandwich ELISA and RT-PCR for merbecovirus detection.

Parameter	Sandwich ELISA	RT-PCR
**Cost per test (USD)**	~2–3	~10–15
**Time to result**	~3–4 hours	~5–6 hours
**Technical skill level**	Moderate	High
**Equipment required**	Basic plate reader	Thermocycler + RNA extraction
**Automation potential**	High	Moderate
